# Maternal advanced age, single parenthood, and ART increase the risk of child morbidity up to five years of age

**DOI:** 10.1186/s12887-021-03103-2

**Published:** 2022-01-14

**Authors:** Malin Lindell Pettersson, Marie Bladh, Elizabeth Nedstrand, Agneta Skoog Svanberg, Claudia Lampic, Gunilla Sydsjö

**Affiliations:** 1 Department of Obstetrics and Gynecology, and Department of Biomedical and Clinical Sciences, Linköping University, SE-581 85 Linköping, Sweden; 2 Department of Women’s and Children’s Health, Uppsala University, Uppsala, Sweden; 3 Department of Women’s and Children’s Health, Karolinska Institutet, Stockholm, Sweden; 4 Department of Psychology, Umeå University, Umeå, Sweden

**Keywords:** Child morbidity, Advanced maternal age, Single status, Assisted reproductive technology

## Abstract

**Background:**

Advanced maternal age, single status and use of assisted reproductive technology (ART) are increasing in mothers in high-income countries, and all are known risk factors for negative obstetric outcomes. Less is known about their long-term consequences for childhood morbidity. Thus, the aim of this study was to investigate morbidity up to five years of age, in the children of older, single, and/or ART-treated mothers.

**Methods:**

A cross-sectional using Swedish registers was performed comprising 23 772 children. The prevalence of diagnosis and the number of hospital visits for specialist care, were compared and analyzed in relation to maternal age at childbirth, maternal civil status, and mode of conception. The odds ratio for specialized care within each ICD-chapter were estimated using single and multiple logistic regression.

**Results:**

Children born to single mothers and children conceived using ART had significantly more outpatient visits for specialist care and significantly more diagnoses compared to children with married/cohabiting mothers, and spontaneously conceived children. Children born to mothers of advanced maternal age (≥40) had fewer in- and outpatient visits. However, they were significantly more often diagnosed within ICD-chapters XVI, XVII i.e., they experienced more morbidity in the neonatal period.

**Conclusion:**

The results indicate that children born to single mothers and children of ART-treated mothers have a higher morbidity and consume more specialist care than children of married/cohabiting and spontaneously pregnant mothers. We conclude that the use of ART, maternal single status and advanced maternal age are risk factors of importance to consider in pediatric care and when counseling women who are considering ART treatment.

## Background

In the Western world, an increasing number of children are being born to mothers of advanced age, i.e., women over 35-40 years old. In Sweden, the average age when giving birth for the first time has increased by more than four years since 1973, and the same is true for other developed countries [1, 2]. This can partly be explained by increasing use of assisted reproductive technology (ART). Advanced maternal age increases the risk for both mother and child with respect to pregnancy and birth-related complications i.e., preeclampsia, gestational diabetes, stillbirth, preterm birth (PT), low birth weight (LBW), small for gestational age children (SGA), and lower Apgar scores [1, 3–5]. ART procedures are also associated with adverse obstetric outcomes for mother and child compared with mothers who conceive spontaneously and their children [6]. A systematic review of child morbidity in relation to ART procedures showed inconsistency but concluded that there may be an increased risk for somatic morbidity in childhood when children are conceived using ART [6, 7].

Mothers of advanced age exhibit a higher morbidity both prior to as well as after childbirth (e.g. infections, neoplasms, endocrine, respiratory or genitourinary issues) [8]. A higher proportion of mothers of advanced age compared with younger mothers are more often single and have used ART e.g. IVF with their own gametes or donated gametes, to conceive [8]. Mothers aged 40-45 years or more can be assumed to have used donated oocytes as well as donated embryos to a greater extent, which is considered a risk factor during pregnancy and childbirth [9–13], but may also have an influence on the child’s health in both the long- and short-term perspectives [10, 12].

Moreover, previous studies have shown that children born to mothers of advanced age have an increased risk of being born with non-optimal birth characteristics, i.e. small for gestational age (SGA), born preterm (PT) and low birth weight (LBW) and with major congenital anomalies, which are all risk factors for future morbidity, in both childhood and adulthood [4, 14–18]. In a recent Danish cohort study, with an average follow-up time of 11 years, it was found that children born to mothers of advanced age, defined as 35 years or older, had a higher overall morbidity compared with children born to mothers aged 25-29 years old [19].

Existing studies primarily focus on cohorts and often limit the results to either advanced age, single motherhood, or mode of conception. To our knowledge, no previous study has examined child morbidity and specialist health care consumption related to maternal age, maternal civil status and use of ART in the same population of children up to five years of age.

The aim of the present study is therefore to investigate to what extent the mothers’ age, civil status and mode of conception affects the child’s morbidity up to five years of age.

Thus, our hypothesis was that advanced maternal age, being a single mother, and the use of ART are associated with a higher morbidity among children up to five years of age.

## Methods

The current study population originated from a larger study comprising all mothers aged 40 years or older (*n* = 37558) who had given birth in Sweden between 2007 and 2012, and a control group of mothers, matched on parity, aged 39 years or younger (*n* = 71 472) [3]. The only exclusion criterion was to not allow any of the index mothers to serve as a control to themselves.

For the present cross-sectional study only children born during 2007-2008 were included to allow for a follow-up on the children’s morbidity up to five years of age. In total, the study population consisted of 23 772 children. Children born to mothers 40 years or older, *n* = 8 203 (34.5%), formed the index group and children born to mothers 39 years or younger, *n* = 15 569 (65.5%), formed the control group.

### Registers

The Swedish Medical Birth Register (MBR) and The National Patient Register (NPR) are both held by the Swedish National Board of Health and Welfare. The MBR includes standardized medical information from the prenatal, delivery and the neonatal care [20] pertaining to almost all births (a small percent – 0.5-3.9 % of all infants are missing in the register) since 1973 [21]. The MBR has been evaluated and deemed suitable for evaluating child health outcomes such as birthweight and perinatal survival. However, evaluation of long-term trends may be difficult due to varying and inexact diagnostic criteria in clinical settings [22]. Also, data loss related to infant diagnoses has been reported and supplementary information from hospital discharge registers is recommended [21]. However, the proportion of missing values on birthweight and gestational age is very low at 0.1 percent. Also, in the current study the infant diagnoses are supplemented using the NPR. The NPR contains patient data, geographical data, administrative data, and medical data pertaining to both inpatient care (since 1964) and specialist outpatient care (since 2001). The quality of the register has been externally reviewed and evaluated, and the NPR is considered to have a good coverage, especially the inpatient part of the register where 99% of the hospital discharges are registered [23].

### Diagnoses

Diagnoses included in the study were retrieved from the International Classification of Disease (ICD) version 10. Chapters of interest were I-XIV and XVI-XX. Chapter XV was excluded since it pertains to the mother’s diagnoses during pregnancy and childbirth, while Chapter XX was excluded because too few subjects were diagnosed within that chapter (Table 1).

### Definitions

Morbidity in children from the neonatal period up to five years of age was investigated by the number of incidences of diagnoses within each of the chapters I- XX (excluding chapter XV) defined in the International Classification of Diseases, version 10 (ICD-10). The diagnoses were retrieved from specialist care unit visits as present in the NPR. Indicator variables for each of the ICD-10 chapters included in the study were defined as either having no chapter-specific visits or having one or more chapter-specific visits. Also, for outpatient visits, all diagnoses (chapters I-XX, excluding chapter XV) were summarized into an indicator of any type of disease defined as “Having zero to ten chapter-specific diagnoses” and “Having 11 or more chapter-specific diagnosis”. Similarly, inpatient visits were summarized and categorized into “Having zero to one chapter-specific diagnoses” and “Having two or more chapter-specific diagnoses”. Any morbidity was defined as the presence of any diagnosis within any of the chapters I to XIX, coded as “yes” or “no”. A modified version of this variable, Any morbidity excluding XVI, was coded similarly.

Maternal age when giving birth was categorized into two levels, ≤39 years of age”, ≥40 years of age”. These will be referred to as “younger mothers” and “older mothers”, respectively. Subgroup analyses were made based on maternal age when giving birth but also with regard to maternal use of ART categorized as either “No” or “Yes” and maternal civil status defined as “Single” or “Married/cohabiting”. Furthermore, demographic characteristics were maternal educational level, defined as either “Elementary”, “High school” or “College/University” registered at the time of childbirth, and maternal nicotine use during pregnancy.

Gestational age was divided into preterm (<37 gestational weeks), and term (≥37 gestational weeks), while birthweight was categorized into “low birthweight” (<2,500 grams) and “normal birthweight” (≥2,500 grams). Lastly, “small for gestational age” (SGA) was defined as a gender-specific birth weight <2 standard deviations (SD) of the mean birth weight for the gestational length, while “large for gestational age” (LGA) was defined as gender-specific birth weight >2 SD. Appropriate for gestational age (AGA) defined those children who were not SGA or LGA [24].

### Statistical analyses

Data were presented as total numbers (n) and percentages (%). Categorical data were analyzed using Pearson’s Chi square statistics and Mann-Whitney U-test was used for continuous data. Multiple logistic regression models estimating the odds ratio (OR) were performed separately for each hospital visit variable (i.e., in total 22 different models). Exposures in the analyses were maternal age, civil status, and ART whereas the independent variables were gender, size for gestational age, gestational age, birthweight, maternal nicotine use during pregnancy and maternal education. In interaction effect defined as a combination of use of ART and maternal age (“No ART and Age ≤ 39”, “No ART and Age ≥40”, “ART and age≤39” and “ART and Age ≤ 39”) was evaluated. All analyses were performed using SPSS, version 26 (IBM Inc., Armonk, NY, USA). Statistical significance was defined as *P* <.05 (two-sided).

### Ethical approval

The study was approved by the Regional Ethical Review Board, Linköping, Sweden and was performed accorded to the Declaration of Helsinki. No 2014/111-31. Date 26-03-2016.

## Results

### Univariate and bivariate analyses

Thirteen percent of the mothers in the study population were single. It was more common among older mothers to be single compared with younger mothers (16% vs 11%). (Table 2). Also, older mothers more often had a college degree (55% vs 47%) and had more often used ART to become pregnant (9% vs 3%) compared with younger mothers. Older mothers were also somewhat more likely to have given birth to a child born PT (7% vs 6%), LBW (5% vs 4%), and/or SGA (3% vs 2%) compared with younger mothers (Table 2). 0.1 % of the population were younger than 18 years and 1% (approximately 110 women) were older than 45 years (Data not shown).

**Table 1:** Description of the included ICD-10 chapters

ICD-chapter	ICD-10 code
I Certain infectious and parasitic diseases	A00-B99
II Neoplasms	C00-D48
III Diseases of the blood and blood-forming organs and certain disorders involving the immune mechanism	D50-D89
IV Endocrine, nutritional and metabolic diseases	E00-E90
V Mental and behavioral disorders	F00-F99
VI Diseases of the nervous system	G00-G99
VII Diseases of the eye and adnexa	H00-H59
VIII Diseases of the ear and mastoid process	H60-H95
IX Diseases of the circulatory system	I00-I99
X Diseases of the respiratory system	J00-J99
XI Diseases of the digestive system	K00-K93
XII Diseases of the skin and subcutaneous tissue	L00-L99
XIII Diseases of the musculoskeletal system and connective tissue	M00-M99
XIV Diseases of the genitourinary system	N00-N99
XV Pregnancy, childbirth and the puerperium	O00-O99
XVI Certain conditions originating in the perinatal period	P00-P96
XVII Congenital malformations, deformations and chromosomal abnormalities	Q00-Q99
XVIII Symptoms, signs and abnormal clinical and laboratory findings, not elsewhere classified	R00-R99
XIX Injury, poisoning and certain other consequences of external causes	S00-S99 T00-T98

**Table 2:** Children born between 2007-2008, maternal demographic data, birth characteristics and medical care

	Total	Maternal age		
	***n*** =23 772	≤39 years ***n*** = 15 569	≥40 years ***n*** = 8203	
	n (%)	n (%)	n (%)	***p***-value
Maternal age				**NA**
≤39 years	15 569 (65.5)	-	-	
≥40 years	8203 (34.5)	-	-	
Civil status				**<.001**
Married/cohabiting	20 767 (87.4)	13 877 (89.1)	6890 (84.0)	
Single status at registration	3005 (12.6)	1692 (10.9)	1313 (16.0)	
Maternal educational level				**<.001**
Elementary	2611 (11.2)	1948 (12.7)	663 (8.3)	
High school	6245 (40.7)	6245 (40.7)	2951 (36.8)	
College/University	7156 (46.6)	7156 (46.6)	4400 (54.9)	
Nicotine use during pregnancy				**<.001**
No	20 289 (90.0)	13 268 (89.5)	7021 (91.2)	
Yes	2242 (10.0)	1564 (10.5)	678 (8.8)	
Gender				**.772**
Boy	12 361 (52.0)	8085 (51.9)	4276 (52.1)	
Girl	11 411 (48.0)	7484 (48.1)	3927 (47.9)	
Assisted reproductive technology^a^				**<.001**
No	22 577 (95.0)	15 095 (97.0)	7482 (91.2)	
Yes	1195 (5.0)	474 (3.0)	721 (8.8)	
Gestational age				**.020**
Born at term	22 185 (93.4)	14 573 (93.7)	7612 (92.9)	
Born preterm^b^	1570 (6.6)	986 (6.3)	584 (7.1)	
Birthweight				**.013**
Normal birthweight	22 611 (95.3)	14 846 (95.5)	7765 (94.8)	
Low birthweight^c^	1122 (4.7)	696 (4.5)	426 (5.2)	
Size for gestational age				**<.001**
Average for gestational age	22 273 (93.7)	15 288 (98.2)	7978 (97.3)	
Small for gestational age	506 (2.1)	281 (1.8)	225 (2.7)	
Hospitalizations				
Outpatient (median/IQR)	2.00/5.00	2.00/5.00	2.00/5.00	**.114**
Inpatient (median/IQR)	0.00/1.00	0.00/1.00	0.00/1.00	**.007**
Total (median/IQR)	3.00/6.00	3.00/6.00	3.00/5.00	**.041**
Outpatient visits				**.743**
0-10	21 046 (88.6)	13 776 (88.5)	7270 (88.6)	
11-	2726 (11.5)	1793 (11.5)	933 (11.4)	
Inpatient visits				**.203**
0-1	20 893 (87.9)	13 653 (87.7)	7240 (88.6)	
2-	2879 (12.1)	1916 (12.3)	963 (11.7)	

^a^ Assisted reproductive technology (ART): ovulation induction, in vitro fertilization (IVF) or intra-plasmic sperm injection (ICSI) with own or donated gametes

^b^ Born preterm (PT): born at <37 weeks’ gestation

^c^ Low birth weight (LBW): birth weight <2500 g

When analyzing morbidity up to five years of age, it was found that children born to younger mothers were more likely to have been given diagnoses pertaining to *Diseases of the ear and mastoid process*, *Diseases of the respiratory system* and *Injury, poisoning and certain other consequences of external causes* compared with children of older mothers (Table 3). Children of younger mothers were also more likely to have *Any type of morbidity* when including as well as excluding *Certain conditions originating in the perinatal period* (Table 3) compared with children of older mothers. Children born to older mothers were more likely to have been diagnosed with conditions listed in *Certain conditions originating in the perinatal period* and *Congenital malformations, deformations and chromosomal abnormalities* (Table 3)*.*

**Table 3:** All children’s diagnoses for each ICD 10 chapter and presented by maternal age, use of ART and civil status

	Maternal age when born			Conceived by ART^a^			Single		
	≤ 39 years	≥40 years		No	Yes		No	Yes	
	n (%)	n (%)	***P*** value	n (%)	n (%)	***P*** value	n (%)	n (%)	***P*** value
I Certain infectious and parasitic diseases			.506			.001			<.001
No	11 264 (72.3)	5968 (72.8)		16 418 (72.7)	814 (68.1)		15 135 (72.9)	2097 (69.8)	
Yes	4305 (27.7)	2235 (27.2)		6159 (27.3)	381 (31.9)		5632 (27.1)	908 (30.2)	
II Neoplasms			.958			.160			.146
No	15 388 (98.8)	8107 (98.8)		22 319 (98.9)	1176 (98.4)		20 533 (98.9)	2962 (98.6)	
Yes	181 (1.2)	96 (1.2)		258 (1.1)	19 (1.6)		234 (1.1)	43 (1.4)	
III Diseases of the blood and blood-forming organs and certain disorders involving the immune mechanism			.394			.064			.665
No	15 411 (99.0)	8110 (98.9)		22 345 (99.0)	1176 (98.4)		20 550 (99.0)	2971 (98.9)	
Yes	158 (1.0)	93 (1.1)		232 (1.0)	19 (1.6)		217 (1.0)	34 (1.1)	
IV endocrine, nutritional and metabolic diseases			.082			.521			.830
No	15 087 (96.9)	7982 (97.3)		21 913 (97.1)	1156 (96.7)		20 151 (97.0)	2918 (97.1)	
Yes	482 (3.1)	221 (2.7)		664 (2.9)	39 (3.3)		616 (3.0)	87 (2.9)	
V Mental and behavioural disorders			.151			.129			.017
No	15 138 (97.2)	7949 (96.9)		21 935 (97.2)	1152 (96.4)		20 189 (97.2)	2898 (96.4)	
Yes	431 (2.8)	254 (3.1)		642 (2.8)	43 (3.6)		578 (2.8)	107 (3.6)	
VI Diseases of the nervous system			.551			.730			.206
No	15 228 (97.8)	8033 (97.9)		22 090 (97.8)	1171 (98.0)		20 330 (97.9)	2931 (97.5)	
Yes	341 (2.2)	170 (2.1)		487 (2.2)	24 (2.0)		437 (2.1)	74 (2.5)	
VII Diseases of the eye and adnexa			.183			.045			.126
No	14 111 (90.6)	7391 (90.1)		20 441 (90.5)	1061 (88.8)		18 807 (90.6)	2695 (89.7)	
Yes	1458 (9.4)	812 (9.9)		2136 (9.5)	134 (11.2)		1960 (9.4)	310 (10.3)	
VIII Diseases of the ear and mastoid process			.001			.172			.432
No	12 311 (79.1)	6637 (80.9)		18 014 (79.8)	934 (78.2)		16 569 (79.8)	2379 (79.2)	
Yes	3258 (20.9)	1566 (19.1)		4563 (20.2)	261 (21.8)		4198 (20.2)	626 (20.8)	
IX Diseases of the circulatory system			.650			.720			.865
No	15 454 (99.3)	8138 (99.2)		22 405 (99.2)	1187 (99.3)		20 609 (99.2)	2983 (99.3)	
Yes	115 (0.7)	65 (0.8)		172 (0.8)	8 (0.7)		158 (0.8)	22 (0.7)	
X Diseases of the respiratory system			<.001			.031			.930
No	9281 (59.6)	5150 (62.8)		13 741 (60.9)	690 (57.7)		12 609 (60.7)	1822 (60.6)	
Yes	6288 (40.4)	3053 (37.2)		8836 (39.1)	505 (42.3)		8158 (39.3)	1183 (39.4)	
XI Diseases of the digestive system			.807			<.001			.051
No	13 715 (88.1)	7235 (88.2)		19 936 (88.3)	1014 (84.9)		18 334 (88.3)	2616 (87.1)	
Yes	1854 (11.9)	968 (11.8)		2641 (11.7)	181 (15.1)		2433 (11.7)	389 (12.9)	
XII Diseases of the skin and subcutaneous tissue			.281			.371			.628
No	13 601 (87.4)	7206 (87.8)		19 771 (87.6)	1036 (86.7)		18 185 (87.6)	2622 (87.3)	
Yes	1968 (12.6)	997 (12.2)		2806 (12.4)	159 (13.3)		2582 (12.4)	383 (12.7)	
XIII Diseases of the musculoskeletal system and connective tissue			.354			.699			.218
No	14 964 (96.1)	7864 (95.9)		21 683 (96.0)	1145 (95.8)		19 930 (96.0)	2898 (96.4)	
Yes	605 (3.9)	339 (4.1)		894 (4.0)	50 (4.2)		837 (4.0)	107 (3.6)	
XIV Diseases of the genitourinary system			.894			.305			.398
No	14 718 (94.5)	7758 (94.6)		21 354 (94.6)	1122 (93.9)		19 625 (94.5)	2851 (94.9)	
Yes	851 (5.5)	445 (5.4)		1223 (5.4)	73 (6.1)		1142 (5.5)	154 (5.1)	
XVI Certain conditions originating in the perinatal period			.002			<.001			.063
No	14 080 (90.4)	7315 (89.2)		20 371 (90.2)	1024 (85.7)		18 719 (90.1)	2676 (89.1)	
Yes	1489 (9.6)	88 (10.8)		2206 (9.8)	171 (14.3)		2048 (9.9)	329 (10.9)	
XVII Congenital malformations, deformations and chromosomal abnormalities			<.001			.021			.065
No	14 123 (90.7)	7324 (89.3)		20 392 (90.3)	1055 (88.3)		18 764 (90.4)	2683 (89.3)	
Yes	1446 (9.3)	879 (10.7)		2185 (9.7)	140 (11.7)		2003 (9.6)	322 (10.7)	
XVIII Symptoms, signs and abnormal clinical and laboratory findings, not elsewhere classified			.468			<.001			.005
No	11 573 (74.3)	6133 (74.8)		16 869 (74.7)	837 (70.0)		15 531 (74.8)	2175 (72.4)	
Yes	3996 (25.7)	2070 (25.2)		5708 (25.3)	358 (30.0)		5236 (25.2)	830 (27.6)	
XIX Injury, poisoning and certain other consequences of external causes			.001			.921			.089
No	11 434 (73.4)	6182 (75.4)		16 729 (74.1)	887 (74.2)		15 351 (73.9)	2265 (75.4)	
Yes	4135 (26.6)	2021 (24.6)		5848 (25.9)	308 (25.8)		5416 (26.1)	740 (24.6)	
Outpatient visits			0.743			0.041			0.004
0-10	13776 (88.5)	7270 (88.6)		20010 (88.6)	1036 (86.7)		18433 (88.8)	2613 (87.0)	
11-	1793 (11.5)	953 (11.4)		2567 (11.4)	159 (13.3)		2334 (11.2)	392 (13.0)	
Inpatient visits			0.203			0.194			0.717
0-1	13653 (87.7)	7240 (88.3)		19857 (88.0)	1036 (86.7)		18258 (87.9)	2635 (87.7)	
2-	1916 (12.3)	964 (11.7)		2720 (12.0)	159 (13.3)		2509 (12.1)	370 (12.3)	
ANY MORBIDITY^b^			.004			<.001			.625
No	3222 (20.7)	1831 (22.3)		4858 (21.5)	195 (16.3)		4404 (21.2)	649 (21.6)	
Yes	12 347 (79.3)	6372 (77.7)		17 719 (78.5)	1000 (83.7)		16 363 (78.8)	2356 (78.4)	
ANY MORBIDITY^b^ excl chap XVI			.002			<.001			.924
No	3433 (22.2)	1954 (23.8)		5181 (22.9)	206 (17.2)		4704 (22.7)	683 (22.7)	
Yes	12 136 (77.9)	6249 (76.2)		17 369 (77.1)	989 (82.8)		16 063 (77.3)	2322 (77.3)	

^a^ Assisted reproductive technology (ART): ovulation induction, in vitro fertilization (IVF) or intracytoplasmic sperm injection (ICSI) with own or donated gametes, embryo donation

^b^ Any morbidity: the presence of any diagnosis within any of ICD-10 chapters I to XIX

Children conceived by ART were more likely to have been diagnosed within several chapters compared with spontaneously conceived children. In addition, children conceived by ART were overrepresented in the summary variable *Any morbidity* (both when including and excluding *Certain conditions originating in the perinatal period*) compared with children spontaneously conceived (Table 3)*.*

Children born to single mothers were more likely to have been diagnosed within for instance *Certain infectious and parasitic diseases, Mental and behavioral disorders* when compared with children born to mothers who were married/cohabiting (Table 3)*.*

## Multivariate analysis of morbidity up to 5 years of age

### Maternal factors

Children born to older mothers were less likely to be diagnosed with any morbidity, both when excluding and including chapter XVI *Certain conditions originating in the perinatal period,* (aOR=0.88, 95% CI=0.82-0.94 in both instances) (Table 4).

**Table 4:** Associations^a^ between Maternal and birth characteristics and the children’s in- and outpatient visits separately, and any morbidity both including and excluding chapter XVI Certain conditions originating in the perinatal period

	Outpatient visits 0-10 vs 11- visits	Inpatient visits 0-1 vs 2 visits	Any Morbidity^b^ (incl chap XVI)	Any Morbidity^b^ (excl chap XVI)
	aOR (95% CI)	aOR (95% CI)	aOR (95% CI)	aOR (95% CI)
Maternal age				
≤39 years	*Reference*	*Reference*	*Reference*	*Reference*
≥40 years	0.97 (0.89-1.06)	0.93 (0.85-1.02)	**0.88 (0.82-0.94)**	**0.88 (0.82-0.94)**
Civil status				
Married/ cohabiting	*Reference*	*Reference*	*Reference*	*Reference*
Single status at registration	1.09 (0.94-1.27)	0.96 (0.82-1.12)	1.05 (0.93-1.19)	1.06 (0.93-1.19)
Assisted reproductive technology^c^				
No	*Reference*	*Reference*	*Reference*	*Reference*
Yes	1.18 (0.98-1.41)	0.95 (0.79-1.15)	**1.39 (1.18-1.64)**	**1.46 (1.24-1.71)**
Gender				
Boy	*Reference*	*Reference*	*Reference*	*Reference*
Girl	**0.66 (0.60-0.72)**	**0.64 (0.59-0.70)**	**0.72 (0.67-0.77)**	**0.72 (0.68-0.77)**
Size for gestational age				
Average for gestational age	*Reference*	*Reference*	*Reference*	*Reference*
Small for gestational age	1.18 (0.91-1.53)	1.11 (0.86-1.43)	1.03 (0.78-1.34)	0.99 (0.77-1.27)
Gestational age				
Born at term	*Reference*	*Reference*	*Reference*	*Reference*
Born preterm^d^	**1.82 (1.51-2.19)**	**3.31 (2.81-3.90)**	**1.98 (1.60-2.45)**	**1.41 (1.17-1.70)**
Birthweight				
Normal	*Reference*	*Reference*	*Reference*	*Reference*
Low birth weight^e^	**1.74 (1.39-2.18)**	**2.06 (1.68-2.53)**	**1.48 (1.13-1.94)**	1.17 (0.93-1.48)
Nicotine use during pregnancy				
No	*Reference*	*Reference*	*Reference*	*Reference*
Yes	**1.37 (1.20-1.56)**	**1.17 (1.03-1.34)**	**1.20 (1.07-1.35)**	**1.22 (1.09-1.38)**
Educational level				
Elementary	**1.31 (1.15-1.50)**	**1.28 (1.11-1.46)**	**1.28 (1.14-1.44)**	**1.30 (1.16-1.45)**
High school	**1.14 (1.04-1.24)**	**1.13 (1.03-1.24)**	1.05 (0.98-1.13)	1.04 (0.97-1.12)
College/ University	*Reference*	*Reference*	*Reference*	*Reference*

^a^ Adjusted for all variables presented in the table. Statistically significant ORs are presented in bold text

^b^ Any morbidity: the presence of any diagnosis within any of ICD-10 chapters I to XIX

^c^ Assisted reproductive technology (ART): ovulation induction, in vitro fertilization (IVF) or intracytoplasmic sperm injection (ICSI) with own or donated gametes, embryo donation

^d^ Born preterm (PT): born at <37 weeks’ gestation

^e^ Low birth weight (LBW): birth weight <2500 g

Moreover, children born to older mothers had a decreased likelihood of being diagnosed within the following chapters: *Diseases of the eye and adnexa* (aOR=0.90, 95% CI= 0.84-0.97) (Table 5)*, Diseases of the respiratory system* (aOR=0.87, 95% CI=0.82-0.92), *Injury, poisoning and certain other consequences of external causes* (aOR=0.90, 95%CI=0.84-0.96) (Table 6) compared with children born to younger mothers. However, children of older mothers were more likely to be diagnosed within *Certain conditions originating in the perinatal period* and *Congenital malformations, deformations and chromosomal abnormalities* (aOR=1.15, 95% CI=1.04-1.28 and aOR 1.16 95%, CI=1.05-1.27 respectively) (Table 5) compared with younger mothers.

**Table 5:** Associations^a^ between maternal and birth characteristics and chapter specific morbidity of diagnosis received in specialist in- and outpatient care

	Chapter I	Chapter II	Chapter III	Chapter IV	Chapter V	Chapter VI	Chapter VII	Chapter VIII	Chapter IX
	aOR (95% CI)	aOR (95% CI)	aOR (95% CI)	aOR (95% CI)	aOR (95% CI)	aOR (95% CI)	aOR (95% CI)	aOR (95% CI)	aOR (95% CI)
Maternal age									
≤39 years	*Reference*	*Reference*	*Reference*	*Reference*	*Reference*	*Reference*	*Reference*	*Reference*	*Reference*
≥40 years	0.96 (0.90-1.03)	1.02 (0.78-1.33)	1.12 (0.85-1.47)	0.84 (0.71-1.00)	1.11 (0.94-1.31)	0.92 (0.76-1.13)	1.04 (0.94-1.14)	**0.90 (0.84-0.97)**	1.03 (0.74.1.43)
Civil status									
Married/ cohabiting	*Reference*	*Reference*	*Reference*	*Reference*	*Reference*	*Reference*	*Reference*	*Reference*	*Reference*
Single status at registration	**1.18 (1.06-1.31)**	1.25 (0.82-1.91)	1.07 (0.67-1.71)	0.99 (0.73-1.33)	**1.33 (1.01-1.74)**	1.03 (0.74-1.44)	1.06 (0.90-1.24)	1.02 (0.90-1.15)	1.01 (0.57-1.80)
Assisted reproduction technology^b^									
No	*Reference*	*Reference*	*Reference*	*Reference*	*Reference*	*Reference*	*Reference*	*Reference*	*Reference*
Yes	**1.31 (1.15-1.50)**	1.37 (0.84-2.24)	1.51 (0.93-2.45)	1.13 (0.80-1.59)	1.27 (0.92-1.75)	0.86 (0.56-1.33)	1.14 (0.94-1.39)	1.14 (0.99-1.33)	0.88 (0.43-1.82)
Gender									
Boy	*Reference*	*Reference*	*Reference*	*Reference*	*Reference*	*Reference*	*Reference*	*Reference*	*Reference*
Girl	**0.90 (0.85-0.95)**	0.86 (0.67-1.10)	0.78 (0.60-1.01)	**0.80 (0.68-0.93)**	1.06 (0.90-1.24)	**0.81 (0.68-0.98)**	1.00 (0.91-1.09)	**0.74 (0.69-0.79)**	**0.68 (0.49-0.93)**
Size for gestational age									
Average for gestational age	*Reference*	*Reference*	*Reference*	*Reference*	*Reference*	*Reference*	*Reference*	*Reference*	*Reference*
Small for gestational age	1.23 (1.00-1.52)	1.52 (0.73-3.18)	1.70 (0.83-3.47)	**2.02 (1.31-3.12)**	1.37 (0.84-2.25)	0.96 (0.56-1.65)	1.18 (0.89-1.56)	0.89 (0.70-1.14)	1.83 (0.82-4.11)
Gestational age									
Born at term	*Reference*	*Reference*	*Reference*	*Reference*	*Reference*	*Reference*	*Reference*	*Reference*	*Reference*
Born preterm^c^	**1.29 (1.11-1.50)**	**2.04 (1.23-3.38)**	**2.00 (1.19-3.39)**	**2.07 (1.50-2.86)**	**1.51 (1.05-2.16)**	**2.09 (1.44-3.02)**	**1.56 (1.27-1.92)**	**1.26 (1.06-1.48)**	0.88 (0.41-1.90)
Birthweight									
Normal	*Reference*	*Reference*	*Reference*	*Reference*	*Reference*	*Reference*	*Reference*	*Reference*	*Reference*
Low birth weight^d^	1.09 (0.90-1.32)	0.88 (0.45-1.72)	1.06 (0.54-2.07)	0.86 (0.56-1.32)	1.12 (0.71-1.75)	**1.92 (1.24-2.97)**	**1.71 (1.34-2.19)**	1.17 (0.95-1.44)	2.07 (0.90-4.81))
Nicotine use during pregnancy									
No	*Reference*	*Reference*	*Reference*	*Reference*	*Reference*	*Reference*	*Reference*	*Reference*	*Reference*
Yes	**1.16 (1.05-1.28)**	0.94 (0.62-1.44)	0.88 (0.56-1.39)	0.92 (0.70-1.20)	0.92 (0.69-1.21)	**1.34 (1.01-1.77)**	**1.42 (1.23-1.63)**	**1.32 (1.18-1.46)**	1.01 (0.60-1.71)
Educational level									
Elementary	**1.47 (1.34-1.62)**	1.30 (0.88-1.92)	1.33 (0.89-1.98)	1.21 (0.93-1.56)	0.91 (0.69-1.20)	1.15 (0.86-1.54)	**1.19 (1.03-1.38)**	**1.32 (1.19-1.48)**	1.02 (0.62-1.69)
High school	**1.11 (1.04-1.18)**	0.99 (0.75-1.30)	0.88 (0.66-1.18)	1.07 (0.90-1.27)	0.91 (0.77-1.08)	0.98 (0.80-1.20)	1.02 (0.92-1.13)	**1.13 (1.05-1.21)**	0.86 (0.61-1.21)
College/ University	*Reference*	*Reference*	*Reference*	*Reference*	*Reference*	*Reference*	*Reference*	*Reference*	*Reference*

^a^ Adjusted for all variables presented in the table. Statistically significant ORs are presented in bold text

^b^ Assisted reproductive technology (ART): ovulation induction, in vitro fertilization (IVF) or intracytoplasmic sperm injection (ICSI) with own or donated gametes, embryo donation

^c^ Born preterm (PT): born at <37 weeks’ gestation

^d^ Low birth weight (LBW): birth weight <2500 g

**Table 6:** Associations^a^ between maternal and birth characteristics and chapter specific morbidity of diagnosis received in specialist care unit visits. Continued

	Chapter X	Chapter XI	Chapter XII	Chapter XIII	Chapter XIV	Chapter XVI	Chapter XVII	Chapter XVIII	Chapter XIX
	aOR (95% CI)	aOR (95% CI)	aOR (95% CI)	aOR (95% CI)	aOR (95% CI)	aOR (95% CI)	aOR (95% CI)	aOR (95% CI)	aOR (95% CI)
Maternal age									
<=39 years	*Reference*	*Reference*	*Reference*	*Reference*	*Reference*	*Reference*	*Reference*	*Reference*	*Reference*
>=40 years	**0.87 (0.82-0.92)**	0.96 (0.88-1.05)	0.97 (0.89-1.06)	1.10 (0.95-1.27)	1.02 (0.90-1.16)	1.15 (1.04-1.28)	1.16 (1.05-1.27)	0.96 (0.89-1.02)	0.90 (0.84-0.96)
Civil status									
Married/ cohabiting	*Reference*	*Reference*	*Reference*	*Reference*	*Reference*	*Reference*	*Reference*	*Reference*	*Reference*
Single status at registration	0.98 (0.88-1.08)	1.09 (0.94-1.27)	1.04 (0.90-1.21)	0.86 (0.66-1.12)	0.85 (0.68-1.06)	1.12 (0.94-1.33)	1.14 (0.97-1.33)	1.15 (1.03-1.29)	1.02 (0.91-1.12)
Assisted reproductive technology^b^									
No	*Reference*	*Reference*	*Reference*	*Reference*	*Reference*	*Reference*	*Reference*	*Reference*	*Reference*
Yes	**1.16 (1.02-1.31)**	**1.33 (1.12-1.58)**	1.15 (0.96-1.37)	1.05 (0.78-1.42)	1.16 (0.90-1.49)	1.02 (0.83-1.25)	1.14 (0.94-1.38)	**1.27 (1.11-1.46)**	0.98 (0.85-1.12)
Gender									
Boy	*Reference*	*Reference*	*Reference*	*Reference*	*Reference*	*Reference*	*Reference*	*Reference*	*Reference*
Girl	**0.67 (0.63-0.70)**	**0.85 (0.79-0.93)**	**0.85 (0.78-0.92)**	**0.78 (0.68-0.90)**	**0.80 (0.72-0.90)**	**0.81 (0.73-0.89)**	**0.68 (0.62-0.75)**	**0.83 (0.78-0.88)**	**0.80 (0.75-0.85)**
Size for gestational age									
Average for gestational age	*Reference*	*Reference*	*Reference*	*Reference*	*Reference*	*Reference*	*Reference*	*Reference*	*Reference*
Small for gestational age	0.96 (0.79-1.18)	1.22 (0.93-1.60)	**0.68 (0.48-0.96)**	0.79 (0.47-1.32)	0.69 (0.45-1.09)	**1.67 (1.28-2.17)**	1.22 (0.92-1.62)	1.17 (0.95-1.45)	0.90 (0.72-1.13)
Gestational age									
Born at term	*Reference*	*Reference*	*Reference*	*Reference*	*Reference*	*Reference*	*Reference*	*Reference*	*Reference*
Born preterm^c^	**1.44 (1.25-1.66)**	**1.46 (1.20-1.77)**	1.00 (0.80-1.24)	**1.16 (0.83-1.63)**	1.33 (1.00-1.76)	**7.95 (6.80-9.30)**	**1.64 (1.34-2.00)**	**1.24 (1.06-1.44)**	0.97 (0.82-1.14)
Birthweight									
Normal	*Reference*	*Reference*	*Reference*	*Reference*	*Reference*	*Reference*	*Reference*	*Reference*	*Reference*
Low birthweight^d^	**1.31 (1.09-1.57)**	1.20 (0.94-1.53)	0.92 (0.70-1.22)	1.23 (0.81-1.87)	1.30 (0.92-1.84)	**3.74 (3.06-4.57)**	**1.43 (1.12-1.84)**	**1.35 (1.12-1.64)**	1.12 (0.92-1.37)
Nicotine use during pregnancy									
No	*Reference*	*Reference*	*Reference*	*Reference*	*Reference*	*Reference*	*Reference*	*Reference*	*Reference*
Yes	**1.28 (1.16-1.40)**	**1.14 (1.00-1.31)**	0.94 (0.82-1.08)	1.20 (0.97-1.50)	1.07 (0.89-1.30)	0.97 (0.82-1.14)	**1.18 (1.02-1.37)**	**1.19 (1.18-1.45)**	0.99 (0.89-1.10)
Educational level									
Elementary	**1.34 (1.22-1.46)**	**1.16 (1.01-1.32)**	**1.26 (1.11-1.44)**	1.16 (0.93-1.45)	**1.56 (1.30-1.87)**	1.12 (0.95-1.32)	1.11 (0.96-1.29)	**1.31 (1.18-1.45)**	1.03 (0.93-1.14)
High school	**1.08 (1.02-1.15)**	1.04 (0.95-1.13)	1.09 (1.00-1.19)	1.07 (0.93-1.24)	**1.20 (1.06-1.37)**	**1.14 (1.03-1.27)**	1.10 (1.00-1.21)	**1.14 (1.07-1.22)**	0.96 (0.90-1.03)
College/ University	*Reference*	*Reference*	*Reference*	*Reference*	*Reference*	*Reference*	*Reference*	*Reference*	*Reference*

Adjusted for all variables presented in the table

^a^ Adjusted for all variables presented in the table. Statistically significant ORs are presented in bold text

^b^ Assisted reproductive technology (ART): ovulation induction, in vitro fertilization (IVF) or intracytoplasmic sperm injection (ICSI) with own or donated gametes, embryo donation

^c^ Born preterm (PT): born at <37 weeks’ gestation

^d^ Low birth weight (LBW): birth weight <2500 g

When compared with spontaneously conceived children, children conceived by ART were more likely to be diagnosed with any morbidity, when including as well as excluding *Certain conditions originating in the perinatal period* (Table 4). Moreover, children conceived by ART were more likely to have been diagnosed within the chapters *Certain infections and parasitic disease* (aOR=1.31, 95% CI=1.15-1.50)*, Diseases of the respiratory system* (aOR=1.16, 95% CI=1.02-1.31)*, Diseases of the digestive system* (aOR=1.33, 95% CI=1.12-1.58)*,* compared with spontaneously conceived children (Table 5).

Children of mothers who were single at the time of childbirth were more often diagnosed within chapter I, chapter V (Table 5), chapter XVIII (all p-values <0.05) (Table 6).

To further elucidate the combined effect maternal age and the use of ART has on the morbidity of the child, models including a combined variable of these two variables were evaluated (no ART and being <40 years of age was defined as the reference category). These analyses did not reveal a clear dose-response effect. However, the analyses showed that being ≥40 years of age and not having used ART decreased the likelihood of the child being diagnosed within chapters VIII, X. and XIX, while increased the likelihood of the child being diagnosed withing chapter XVII compared to women <40 years of age who had conceived spontaneously (data not shown). Moreover, children of women who had used ART to conceive had an increased likelihood of being diagnosed within chapters I, XI, XVII, and XVIII. The analyses also showed that children to women aged ≥40 years who had used ART to conceive had an increased likelihood of being diagnosed within chapter I, compared to children to women <40 years of age who had conceived spontaneously (data not shown).

### Birth characteristics

The multivariate analyses revealed that children of female gender had significantly fewer out- and inpatient visits (aOR=0.66, and aOR=0.64, respectively) (Table 4) and were less likely to have been diagnosed within all but four chapters in the ICD-10 (Table 5 and Table 6) than male children.

Furthermore, PT children were more than three times more often categorized as having had inpatient visits aOR=3.31, 95% CI=2.81-3.90 and were nearly twice as often diagnosed with any type of disease (*Any Morbidity*) compared with children born at term aOR=1.98, 95% CI=1.60-2.45 (Table 4). Preterm children were more likely to have been diagnosed within all but four of the studied ICD chapters.

Also, children born with LBW were twice as often categorized as having had specialist inpatient care. Children with LBW were more likely to have been diagnosed with any somatic diagnosis including *Certain conditions originating in the perinatal period* (Table 4). This increased risk was mainly due to the threefold increased risk of being diagnosed within *Certain conditions originating in the perinatal period* compared with children of normal birth weight (aOR=3.73, 95% CI=3.06-4.57) (Table 5).

Being born SGA did not, for the most part, increase the risk of being diagnosed. Exceptions were *Endocrine, nutritional and metabolic diseases* and *Certain conditions originating in the perinatal period* where children born SGA had an increased likelihood of being diagnosed compared with AGA children.

All models were evaluated when including twinning and maternal preeclampsia/eclampsia. However, including these variables did not alter any of the estimated odds ratios of being diagnosed within each chapter for either maternal age, use of ART, or civil status (data not shown). Thus, the simplified models were preferred to those including twinning and preeclampsia/eclampsia.

## Discussion

The results indicate that morbidity in children up to five years of age is associated with the mode of conception, civil status, and maternal age at time of childbirth.

Children born after ART were more often diagnosed within several chapters, were more often born with non-optimal birth characteristics and more in- and outpatient visits in specialist health care, when compared with spontaneously conceived children.

Children of single mothers had significantly more often been diagnosed within the chapter V *mental and behavior disorders*, chapter XVIII *symptoms, signs and abnormal clinical and laboratory findings* and *certain infectious and parasitic diseases*. Children of single mothers were found to be either equally or more often diagnosed within all chapters of ICD-10, though not always reaching statistical significance, compared with married/cohabiting mothers.

Adverse perinatal outcomes often have long-term health effects for children [25–31]. ART procedures such as in vitro fertilization (IVF) and intracytoplasmic sperm injection (ICSI) are associated with an increased risk of negative obstetric and perinatal outcome in singleton pregnancies when compared with spontaneous conception [32–34]. Moreover, singleton pregnancies after oocyte donation, when compared with IVF with own gametes, are associated with increased obstetric and perinatal risks [35]. Previous studies have reported an increased risk of childhood morbidity and birth defects in ART-conceived children when compared with spontaneously conceived children [7, 36, 37]. ART-conceived children in our study were more likely than spontaneously conceived children to be diagnosed within chapter XVI *Certain conditions originating in the perinatal period*. We followed the children up to five years of age, and ART-conceived children were, overall, more often diagnosed in specialist care. The perinatal and neonatal morbidity aside, children born after ART procedures seem to fare worse throughout childhood and adolescence. Levin et al. (2019) found IVF to be an independent risk factor for long-term pediatric neurologic morbidity, such as attention deficit/hyperactivity disorders, even when obstetric complications were included in the models [38].

To what extent this risk factor actually depends on the ART procedure is debated in two studies by Goisis et al. (2017; 2019) where specifically age- or ART-related associations with the negative birth outcomes, born preterm and born with low birth weight, were statistically and substantially negligible when comparing obstetric outcomes in ART-conceived and naturally conceived children of the same mother [39, 40]. They suggested that other unobserved factors, such as parental underlying health, subfertility, psychological stress or genetic factors, might negatively affect birth outcomes to a larger extent than ART or maternal age per se. We have recently concluded that mothers of advanced age are significantly more often diagnosed within several ICD-10 chapters compared with the younger mothers [41], most likely adding to earlier mentioned risk factors for both neonatal and childhood morbidity.

Children born to single mothers had significantly more diagnoses within three of the ICD-10 chapters. The reason for this is not well understood but single maternal status is negatively related to gestational age and birthweight [3, 42, 43], which are known to have long-term effects on child development and morbidity [25–31]. Our study shows an increased prevalence of diagnoses of mental and behavioral disorders in children born to single mothers. Psychopathology in adolescence has previously been presented [44] but concerning young children this is, to our knowledge, new information.

Previous studies have associated single motherhood with worse mental health, financial hardship, perceived lack of emotional and social support and parental stress when compared with married/cohabiting mothers [45–49] which in turn most likely affects the child as well as the parent. The reason for being single at registration in the prenatal care unit was not defined in our study. One recent study found that children of women who become single mothers by choice, were equal to children in two-parent families in terms of emotional, mental and behavioral adjustment [50]. Poulain et al. (2020) and Crosier et al. (2007) suggest that single status per se might not be the actual risk factor; rather, the single mother’s parenting seems to be compromised by other psychosocial factors (socio-economic status [51], financial stress, less support, non-shared parenting) and mental health problems [52] than the lack of a co-parent, and that these factors are less pronounced in the single mother by choice.

Advanced maternal age was not found to be associated with the number of diagnoses or in- or outpatient visits. This contrast to the results presented in a study by Hviid et al. (2017) where children born to mothers of advanced age (≥35) had a higher overall morbidity compared with children born to younger mothers, although their control group was 25-29 years of age [19]. The control group in the present study ranged from 15 to 39.

However, the findings in the current study are in accordance with a recent study by Pariente et al. (2019) showing no significant difference in morbidity beyond the neonatal period in children of older mothers compared with younger [53]. Since maternal age in most other studies [3, 19] is associated with negative outcomes for both child and mother it is possible that older mothers seek medical care for their children from the primary care services to a greater extent than they seek specialist care. One can also speculate that our results may indicate a higher parity among the older mothers compared with the younger mothers; thus, reflecting more secure mothers, less alarmed by their children’s symptoms and therefore less prone to seek medical attention. This speculation is partly supported by a study where firstborn children were found to have a higher morbidity compared with later born children [54]. Moreover, the results showed that children born to older mothers were more likely to be diagnosed with *Certain conditions originating in the perinatal period* and *Congenital malformations, deformations and chromosomal abnormalities* (chapter XVI and XVII in ICD-10), and to some extent this is supported by a study by Frederiksen et al. (2018) comparing the pregnancy outcomes of mothers of advanced age (≥40) with those of mothers younger than 40. Their study showed a just over threefold risk of increased chromosomal abnormalities but no significant increase in risk of congenital malformations, and they concluded that advanced maternal age has a greater impact on adverse obstetric outcomes [17]. Another study, conducted by Goetzinger et al. (2017) found an overall *decreased* risk for major anomalies in fetuses of mothers of advanced age (≥35) and they suggested an all or nothing phenomena causes their results — that anatomically normal fetuses are more likely to survive since they are developed from younger women’s oocytes [4].

Our results also show that children born SGA, who are more often born to older mothers, had more in- and outpatient visits up to the age of five, compared with children born average for gestational age. This result is in accordance with several previous studies [29–31] showing an increased risk for morbidity within several areas e.g. impaired cognitive development [29], pediatric ophthalmic morbidity [30], long-term pediatric gastrointestinal morbidity [31] in children born SGA.

The threshold ages for a significant increase of negative obstetric and perinatal outcomes for both mother and child are to some extent inconclusive, varying between studies, populations and type of risk considered [55, 56]. However, most results in previous studies point to an increase of risks with higher chronological age.

There are some limitations in the current study. Although there is no recollection bias, there are always some concerns regarding missing data when using register-based data. In the current study, data were collected from 2007 from MBR and NPR. By this year the outpatient data reporting had improved since its introduction in 2001. There are still some issues with missing main diagnosis, to reduce the effect of this problem secondary diagnoses were included in the current study. However, any problems with missing data can be assumed to be random, and thus affect each group in a similar manner. Another problem is the possibility of missing potential confounders in the analyses, i.e., the presence of residual confounding. Unfortunately, this problem can never be completely removed. It is also possible that some residual confounding is present due to categorization of maternal age. In categorizing age, some of the information is lost. However, maternal age was categorized to be in line with the clinical regulations in Sweden, where women are allowed ART treatment until 40 years of age.

The high number of subjects in our study is a great strength, and the national registers containing details of birth data, diagnoses, and visits to specialist health care in Sweden are reliable and validated. We have only used data from the specialist care units and thus capturing the more advanced or pronounced health situation for the children. Another strength of using validated registers is the elimination of e.g. recollection bias.

We have no knowledge of what type of ART the women had used since this was not available in the register. Further analysis of specific ART treatments is warranted and would perhaps help to refine the results.

## Conclusion

The numbers of advanced aged mothers, single mothers and mothers who have used ART are likely to increase further in middle- and high-income countries. With these maternal characteristics, child morbidity may increase as well. The results indicate that children of mothers with single status at pregnancy and children of mothers who have used ART have a higher morbidity and consume more specialist care than married/cohabiting and spontaneously pregnant mothers. We conclude that the use of ART, maternal single status and, in combination with the former factors, advanced maternal age are risk factors that are important to consider in pediatric care and when counseling women considering ART treatment.

## Data Availability

The data in the current study can, due to the legal agreement accepted upon delivery of data from the National Board of Health and Welfare, be only shared on a group level. For requests contact M. Bladh, MD.

## Abbreviations

AGA: Average for gestational age
SGA: Small for gestational age
PT: Born preterm
AT: Born at term
LBW: Low birth weight
ART: Assisted reproductive technology
IVF: In vitro fertilization
